# Force systems and the function of attachments in aligner biomechanics: an analytical approach

**DOI:** 10.1590/2177-6709.31.2.e26spe2

**Published:** 2026-06-22

**Authors:** Wendel SHIBASAKI, Wislei de OLIVEIRA

**Affiliations:** 1 Private practice (Salvador/BA, Brazil).; 2 Private practice (Luis Eduardo Magalhães/BA, Brazil).

**Keywords:** Aligners, Biomechanics, Removable orthodontic appliance, Tooth movement, Alinhadores, Biomecânica, Aparelho ortodôntico removível, Movimentação dentária

## Abstract

**Introduction:**

The growing adoption of aligners contrasts with the lack of clear foundation in the literature regarding their activation methods. Studies often focus on effectiveness or estimated forces, lacking rational standardization and frequently presenting results that conflict with clinical practice.

**Objective:**

This article analyzes the theoretical inconsistencies about how aligners work and proposes an alternative analytical approach to understand the force system they generate. It also explores how the use of attachments can modulate the direction of these forces to enable specific orthodontic movements.

**Methods:**

To assess aligner activation, a two-dimensional model representing a maxillary central incisor was developed. This model, using palatal tipping and extrusion movements, enabled analysis of the aligner’s activation mechanism and the influence of attachments on force redistribution.

**Results:**

The analysis showed consistency with previous studies, providing a rational explanation for aligner activation in these movements. Attachments proved essential in modifying force distribution, enabling displacements beyond the buccolingual axis.

**Conclusions:**

In their current configuration, aligners predominantly generate normal buccal and lingual compressive forces through flexion, lacking intrinsic capacity to produce orthodontically useful forces such as traction, torsion, or shearing in the buccal and lingual planes. However, the strategic use of attachments allows interception of the aligner’s deactivation and redirection of a fraction of the original forces, making other types of orthodontic movements feasible. This highlights the need for a rigorous biomechanical approach for each necessary movement for each tooth, to optimize aligner efficiency in clinical treatments.

## RESUMO

**Introdução:**

A crescente adesão aos alinhadores contrasta com a falta de fundamentação clara na literatura sobre seus métodos de ativação. Estudos focam na eficácia ou em forças estimadas, sem padronização racional, e frequentemente apresentam resultados conflitantes com a prática clínica.

**Objetivo:**

Este artigo analisa as inconsistências teóricas sobre o funcionamento dos alinhadores e propõe uma abordagem analítica alternativa para compreender o sistema de forças gerado. Além disso, explora como a utilização de *attachments* pode modular a direção dessas forças para viabilizar movimentos ortodônticos específicos.

**Métodos:**

Para avaliar a ativação dos alinhadores, foi desenvolvido um modelo bidimensional representando um incisivo central superior. Esse modelo, utilizando os movimentos de inclinação palatina e extrusão, permitiu a análise do mecanismo de ativação dos alinhadores e a influência dos *attachments* na redistribuição das forças.

**Resultados:**

A análise realizada demonstrou coerência com estudos prévios, explicando de forma racional a ativação dos alinhadores nesses movimentos. Os *attachments* se mostraram fundamentais para modificar a distribuição das forças, possibilitando deslocamentos além do eixo vestibulolingual.

**Conclusões:**

Na configuração atual, os alinhadores geram predominantemente forças normais de compressão vestibular e lingual por flexão, sem capacidade intrínseca de produzir forças úteis à Ortodontia por esforço do tipo tração, torção e cisalhamento nos planos vestibular e lingual. No entanto, o uso estratégico de *attachments* permite interceptar a desativação do alinhador e redirecionar uma fração das forças originais, tornando viáveis outros tipos de movimentos ortodônticos. Isso evidencia a necessidade de uma abordagem biomecânica criteriosa em cada movimento necessário a cada dente, para otimizar a eficiência dos alinhadores nos tratamentos clínicos.

## INTRODUCTION

In recent years, the use of orthodontic aligners has significantly increased among orthodontists. Recent studies indicate that approximately 25% of American professionals report using aligners exclusively to treat their patients in clinical practice^1^, and the new generation of orthodontists believes aligners will become the primary technique for treating malocclusions in the future^2^. This may be attributed to the fact that aligners cause less discomfort^3^, are more aesthetically pleasing^4^ ^,^ ^5^, and facilitate eating and oral hygiene^6^ ^,^ ^7^. However, despite their growing use and various modifications aimed at improving these devices, the average effectiveness of aligners remains low, ranging between 41% and 50%^8^ ^,^ ^9^. 

Unlike conventional orthodontics, which is based on the interaction between brackets and archwires^10^ ^,^ ^11^, the interaction between aligners and teeth has not yet been fully described and explained in the literature. A recent systematic review focused on evaluating the force system of aligners^12^ concluded that there is a lack of studies specifically addressing the force system applied by aligners. Most of the existing studies deal with digital planning software, the effectiveness of movements, or estimated force magnitudes obtained through laboratory methods, without rational standardization of how these devices are activated. 

The two most commonly used laboratory methods for evaluating orthodontic forces in aligners are finite element analysis (*in silico*) and the experimental method using nanosensors. The Finite Element Method (FEM) consists of discretizing a continuous system by dividing the structure into small interconnected parts called ‘elements’. These elements are connected at specific points called ‘nodes’, where the interactions between different parts of the structure are calculated^13^. On the other hand, the use of nanosensors, attached to artificial teeth or experimental models, allows direct measurement of the stresses exerted at the contact point with the aligner. These sensors capture the applied forces and transmit data to a software, which displays the forces and moments generated in the three spatial dimensions (3D)^14^. 

Unfortunately, both methods have failed to consistently explain the biomechanical behavior of aligners. Studies using FEM suggest that double attachments increase the effectiveness of aligners for root angulation movements (mesiodistal) of central incisors during diastema closure^15^. Additionally, it is suggested that bodily movement of canines during retraction can be achieved using double or rectangular attachments^16^. However, when these recommendations are applied in clinical practice, the results reveal the ineffectiveness of these accessories: canines tend to tip and rotate distally during retraction with aligners, regardless of the presence of attachments. Moreover, it has been shown that double attachments performed worse in controlling canine root movement^17^. Similarly, studies using sensors face limitations in understanding aligner function. Generally, the artificial teeth used in these experiments are pre-segmented from the rest of the model and attached to the sensor; then the aligner is fully seated, and only afterward the tooth is moved within the aligner, so that forces can be measured. This procedure was used in a study that tested different attachment configurations for premolar rotation correction^18^, whose results suggested better performance with a vertical rectangular attachment. However, the most coherent use of the data from this study may be for analyzing the containment effects of rotation rather than evaluating the effectiveness of aligners in correcting dental rotations, since the study measured resistance to movement. Moreover, clinical studies do not support the recommendation of a superior method for correcting premolar rotations using aligners^19^. 

The study of the mechanical response of aligners can employ experimental methods, but also numerical and/or analytical methods aimed at elucidating the stresses produced by deformation during their potential activations. 

Considering that studies using both methods previously discussed failed to guide clinical practice in predicting the mechanical behavior of aligners, a reanalysis of the theoretical foundation is necessary to understand their interactions with teeth and/or attachments. In this context, the present article proposes an analytical theoretical approach to the force systems generated by aligners during orthodontic movement, exploring the role of attachments as auxiliary elements in redirecting these forces with the aim of expanding the mechanical possibilities of aligners. 

## MATERIAL AND METHODS

### APPLIANCE GEOMETRY AND TOOTH ANATOMY

**Figure 1:**
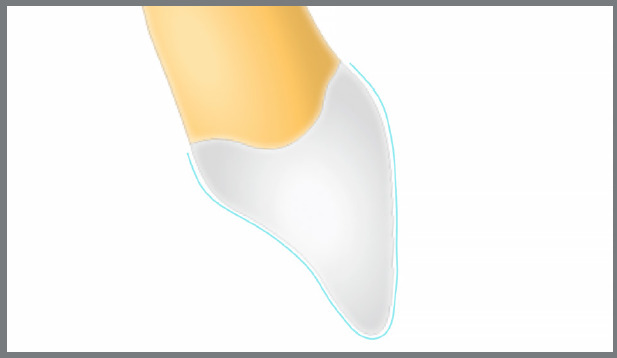
Two-dimensional model in a lateral view of a maxillary central incisor. The blue line represents the aligner’s neutral surface.

To analyze the interaction of aligners with teeth and attachments, a two-dimensional model of a maxillary central incisor was developed (Fig. 1), and evaluated in the movements of palatal tipping and extrusion. These movements were selected for their biomechanical relevance: buccolingual tipping represents the fundamental mechanism of action of aligners, while extrusion is considered the most challenging, as the appliance lacks a cervical surface capable of applying force directly in the occlusal direction. 

### THE PHYSICAL MODEL

**Figure 2:**
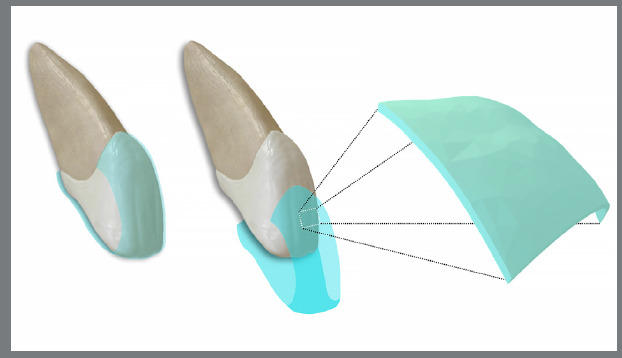
Three-dimensional model of a thermoplastic aligner for a maxillary incisor. Shell-type structures have thickness significantly smaller than the other two dimensions.

The initial step in evaluating the deformations of a structure under a load and identifying the resulting stresses is to conceptualize a physical model that can be analyzed. Orthodontic aligners, for instance, can be represented as shell-type structures (Fig. 2). These structures exhibit curvature in two planes-mesiodistal and cervico-incisal-and have a thickness considerably smaller than the other dimensions, subjected to perpendicular loads to the plane, showing flexural behavior. 

Understanding basic physics concepts is essential to understand how deformation of orthodontic devices occurs, since it is this deformation that generates the force system transmitted to the teeth. This knowledge allows refined control of orthodontic biomechanics, aiding in the anticipation of side effects and in identifying the limitations of each movement system. 

**Figure 3:**
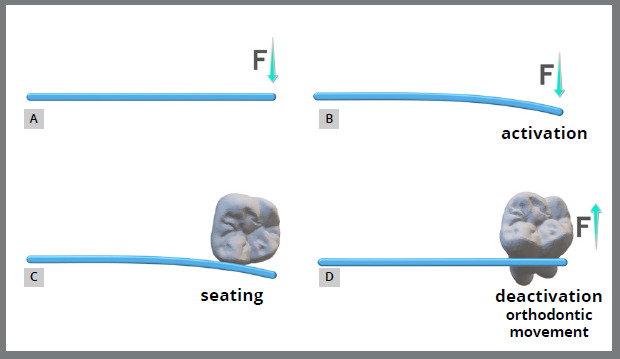
A force applied to any orthodontic appliance (**A**) induces elastic deformation (**B**), activating it. When fitted into the mouth, the teeth experience the deactivation force (**C**), and orthodontic movement occurs (**D**).

From a physical perspective, the active part of any orthodontic appliance can be understood as a spring or a set of springs, which deform under a load (activation force of the appliance) within the elastic range of the material, storing mechanical energy. This energy is then released during the return to its original shape, manifesting as the deactivation force responsible for inducing tooth movement (Fig. 3). Therefore, if the appliance cannot be activated, it will not be deactivated; in other words, if it does not have the capacity to deform and return as closely as possible to its original shape, orthodontic movement will not occur. 

Unlike conventional fixed appliances, aligners have a vertical insertion axis and, even when fabricated passively (without planned movements), require slight temporary deformation until they reach their final position. This deformation capacity enables both the fitting and removal of the aligner, and is essential for executing their activations. 

## ACTIVATIONS

It is precisely at the moment of insertion into the dental arch, in the occlusal-cervical direction, that the activation of orthodontic aligners occurs, increasing the distance between their buccal and lingual surfaces, similar to an interpotent lever, deforming the polymer within its elastic limit. This time, however, the aligner does not return to its original shape as it would when passive, due to the discrepancy between its planned shape and the anatomy of teeth that have not yet been moved, thus remaining deformed. In this way, the aligner conforms to the tooth through this deformation and maintains it until the tooth or teeth with planned movement reach the new desired position. 

The small difference between the aligner’s shape and the anatomy of the tooth to be moved in each setup stage results in a flexural load (Fig. 5). This mechanism generates tooth movement based on the progressive adaptation of the aligner structure, which aims to become passive once the teeth reach their final position. 

[Fig f4]

**Figure 4: f4:**
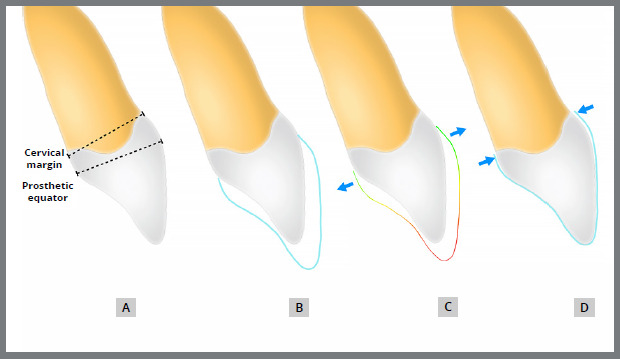
Vertical insertion and deformation of the aligner during passive seating on a tooth without planned movement. **A)** Larger buccolingual dimension at the prosthetic equator than at the cervical margin. **B)** Insertion path obstructed by contact points and the prosthetic equator. **C)** Cervical opening of the aligner and incisal stress area. **D)** Return to the original shape, with full seating.

**Figure 5:**
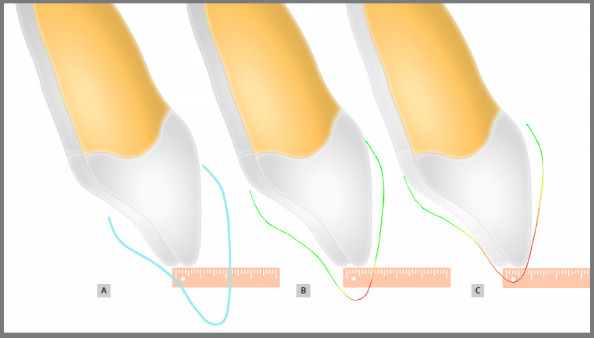
Planned lingual movement (shaded teeth) occurs under the force generated upon aligner seating (**A**), when the buccal surface flexes (**B**) and remains misadapted (**C**), until the orthodontic movement takes place.

Tooth movements performed with aligners, just like in conventional fixed Orthodontics using continuous wires (straight wire), follow principles of an ideal shape-that is, movements are guided by form. 

**Figure 6:**
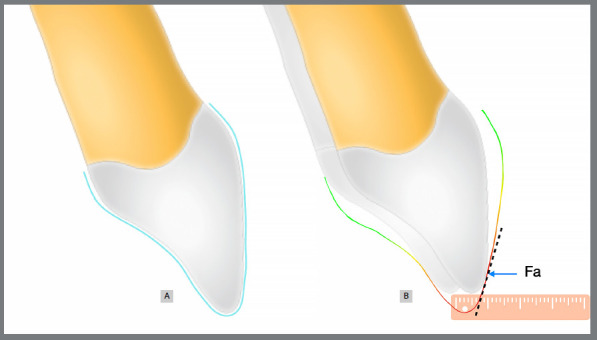
A) Aligner fabricated based on the setup of a palatally tipped incisor. B) Upon seating before movement, only one surface touches the crown, while the opposite surface remains separated, allowing space for the planned displacement. Fa represents the force applied by the aligner.

In the model created for the palatal tipping movement, only one of the surfaces is flexed, based on the aligner’s adaptation to the actual dental arch of the patient. During seating, the buccal surface of the aligner is pressed against the tooth, while the lingual surface remains separated, creating space for the planned displacement. Anchorage is provided by adjacent teeth (Fig. 6). 

After the aligner is seated, the activation force ceases, and the deactivation phase begins, during which the aligner material tends to return to its original shape, releasing the orthodontic forces needed to move the teeth. These forces are predominantly concentrated on the buccal surface, while the mesial and distal surfaces are less involved due to the presence of adjacent teeth, which limit activation. In this process, the incisal edge of the aligner plays a crucial role, concentrating the mechanical effort essential for activation, acting as a spring mechanism. Thus, its integrity is directly related to the generation of forces by the aligner^20^ and to the transmission of these forces to the periodontal ligament, triggering a sequence of biological events that culminate in the desired tooth movement. 

### DISTRIBUTED FORCES APPLIED BY ALIGNERS

**Figure 7:**
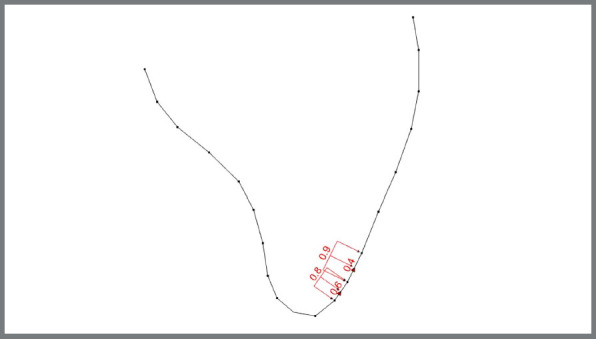
Force distribution, or force action field, for a lingual tipping movement. The doted black line represents the aligner’s neutral axis. The red lines represent the distributed forces and stress values in global and local coordinate systems for a unit load.

To define the force system required for a specific tooth movement, it has always been essential to consider the point of force application. The concept of a concentrated force (force application point) refers to a force applied on a surface whose area is negligible, compared to the other dimensions of the body, which allows this force to be treated as if it were concentrated at a single point. However, when the application area is significant, the force is called distributed, and the variation in force distribution must be considered. This distribution is known as the force action field, which occurs over a surface. The larger the force action field, the lower the stress, resulting in less intense physical strain on the material (Fig. 7). 

The force exerted by the aligner on the tooth can be described as a normal compressive, distributed force, predominantly parallel to the occlusal plane, whose intensity is characterized by normal stress. 

**Figure 8:**
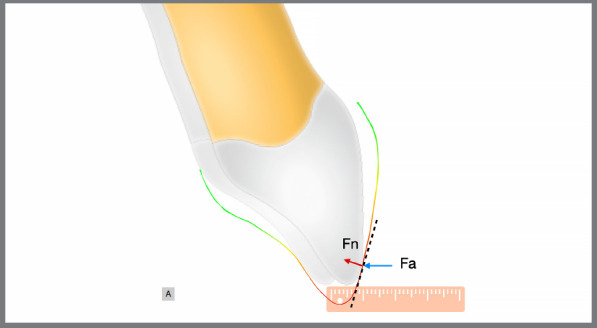
Fa = applied force; Fn = normal force, perpendicular to the contact surface.

**Figure 9:**
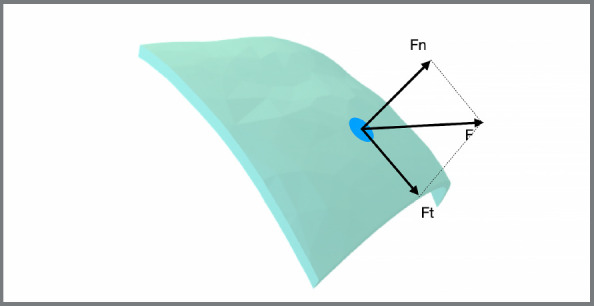
A surface force F decomposed into normal force (Fn) and tangential force (Ft) on an infinitesimal surface area.

Normal forces are reactive forces and always perpendicular to the contact surface (Fig. 8). Thus, the forces exerted by aligners on the dental surface-with a certain curvature radius, or even a flat surface inclined relative to the aligner’s force line-can be decomposed into normal and tangential components (Fig. 9). 

In Orthodontics, to account for the force system applied on a tooth, only the normal force is considered (red arrow in Fig. 8), corresponding to the tooth’s action on the aligner, forming an action-reaction pair. 

A clinically observed effect is intrusive movement, described in the literature^21^ as a side effect of buccal and lingual activation by aligners. This intrusive movement results from the normal forces created by the interaction between the aligner and the dental surfaces. As illustrated in Fig. 8, these normal forces have a resultant with a direction that contributes to the intrusive displacement of the tooth. 

**Figure 10:**
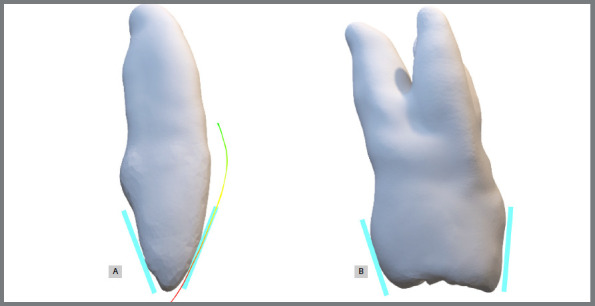
A) Greater divergence between buccal and lingual surfaces in anterior teeth. B) Lesser divergence in posterior teeth.

Therefore, the inclination of the tooth surface can modulate the intensity of the force generated by the aligner in the desired direction. Anatomically, teeth present divergence between the buccal and lingual surfaces, forming an angle relative to the direction of the force applied by aligners. This angle is greater in anterior teeth than in posterior teeth (Fig. 10), which benefits buccolingual movements in the former. 

**Figure 11:**
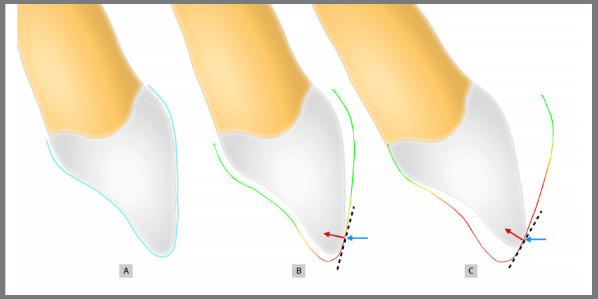
A) An aligner fabricated on a palatally positioned tooth. B) An incisor with reduced initial inclination relative to the aligner activated for palatal tipping movement, resulting in less intrusion, as indicated by the direction of the red arrow. C) An incisor with greater initial inclination relative to the aligner, activated for palatal tipping movement, resulting in greater intrusion.

This difference between the angle of the tooth surface and the aligner can also vary depending on the tooth’s initial inclination, which results in a more incisal contact the greater the buccal inclination of the incisors in a palatal tipping movement^21^ (Fig. 11). 

Therefore, the more parallel the buccal surface of the tooth and the aligner are at the contact point, the more favorable the resulting force will be for palatal tipping movement. 

**Figure 12:**
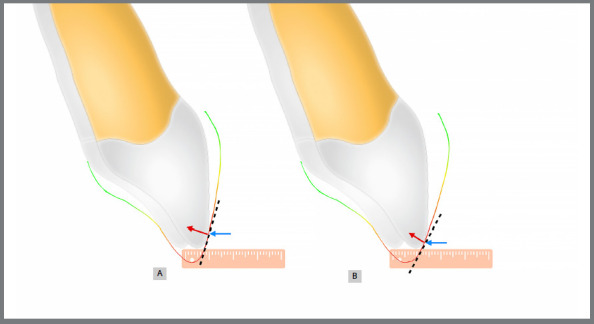
Aligner in **B** was fabricated with twice the activation of the one in **A**. Note that the redirection of the force changes according to the flexion of the buccal surface.

Still analyzing this contact, it is observed that the greater the aligner activation, the greater the misfit produced (Fig. 12)- that is, the discrepancy between the tooth and the aligner-, which alters the direction of forces similarly. This is also supported in the literature^21^. 

This increased activation can be planned intentionally or result from delayed tooth movement, as occurs when adjacent teeth obstruct the movement. In orthodontic appliances, within the material’s limits, the force generated is proportional to the applied activation. However, increasing activation to intensify the force may also alter its direction undesirably. In esthetic aligners, materials are chosen primarily for transparency, favoring polymers, especially thermoplastics, which brings important implications for their mechanical behavior and clinical effectiveness. 

### THERMOPLASTIC MATERIAL

Thermoplastic materials can be defined as linear or branched chain polymeric materials, solid at use temperature-normally ambient or near it-that, when subjected to a substantial increase in temperature (T) and/or pressure (P), soften and flow, allowing them to be molded under such conditions. Once the stimuli (T and P) are removed, they solidify and acquire the shape of the mold^22^. 

**Figure 13:**
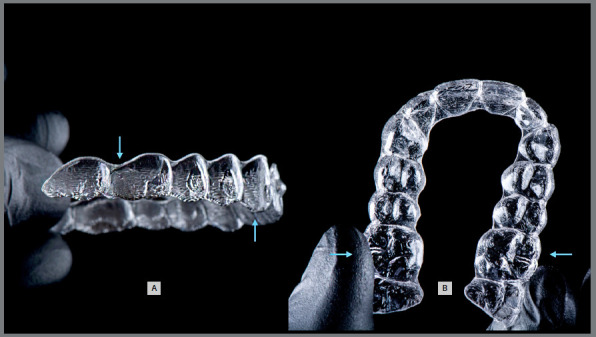
A) High rigidity when attempting vertical activation. B) Greater flexibility in horizontal activation.

The deformation of an orthodontic device made of a homogeneous and isotropic material is accompanied by displacement directly proportional to the applied force, characterizing linear deformation displacement. This relationship between force and displacement defines the rigidity of the material composing the device, known as the elastic constant, or stiffness coefficient. To determine the rigidity of each element, it is necessary to know both the properties of the material used to fabricate the device and the boundary conditions. Aligners, for instance, may be more flexible when activated in specific areas and in a given direction^23^, but may exhibit high rigidity in the same area when the load is applied in another plane, such as the vertical^24^ (Fig. 13). 

There is no doubt that forces are required to induce orthodontic movement. Even though the ideal force magnitude is not a settled issue in the literature^25^, it is known that maintaining active orthodontic force for the necessary time for each movement is essential-that is, keeping the appliance activated. This is why understanding the mechanical properties of the materials used is necessary. 

Two of the most defining characteristics of polymers-critical to understanding their mechanical behavior in Orthodontics-are viscoelasticity and their extreme time-dependence regarding mechanical properties. When a polymeric sample is deformed and kept under constant deformation, the stress needed to maintain this deformation decreases over time. This phenomenon is known as stress relaxation. It explains why aligners lose force after being inserted on the teeth, which do not move immediately, thus keeping the aligner deformed for a time. 

**Figure 14:**
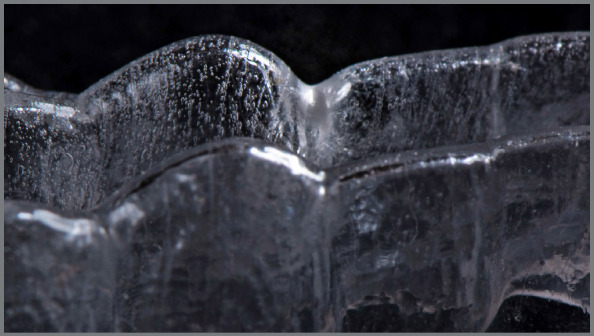
Opacification and localized microcracks in the aligner, in the mesial region of the canine.

Due to their viscoelasticity, we can state that the elastic phase of polymeric materials-during which the material returns to its original shape, once the deforming force ceases-is extremely short, and the material can undergo extended deformation if the force persists^26^ ^,^ ^27^. The low elasticity of the material limits aligner efficiency in movements requiring tooth traction. In such clinical situations, the aligner would need to stretch to allow fitting and then return to its original shape to promote displacement of the target tooth. However, this does not occur; when stretched, the aligner undergoes permanent deformation, a condition clinically manifested by opacification and localized microcracks (Fig. 14). 

Moreover, stress relaxation has great clinical relevance in Orthodontics. This is because the substantial reduction in force level begins within the first minutes of the deactivation phase^24^ and can drop below the ideal range for tooth movement. Thus, maintaining force within the optimal range for longer represents an even greater challenge for these materials. 

**Figure 15:**
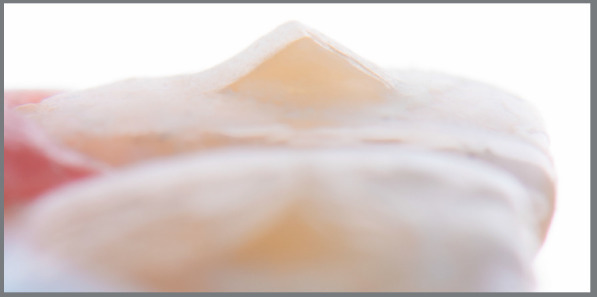
Structure made of composite added to the tooth surface, referred to as an attachment.

So far, it is understood that the direction of the force generated by the aligner can be modified by the difference in inclinations between the aligner and the tooth surface-determined by anatomy, initial inclination, or degree of activation. Since activation is achieved by flexing the buccal and lingual walls, with an incisal fulcrum, the resulting deactivation can only produce forces predominantly in the buccal or lingual directions. In the model proposed in this study, it acts primarily in the palatal direction. However, for movements in other directions, the force produced by aligner deformation can be redirected when transmitted to the teeth, by adding small auxiliary structures made of composite bonded to the tooth surface, called “attachments” (Fig. 15). 

## ATTACHMENTS

Attachments were introduced as a major promise to improve aligner performance, and to this day, considerable responsibility is attributed to them. However, much of what is assumed about their function has never been validated by scientific studies^28^. This may be due to a lack of understanding of their principles, resulting in frustrated expectations caused by improper use of these accessories. 

From a physical standpoint, attachments should be regarded as a simple machine called an inclined plane, which allows the decomposition of the horizontal deactivation force of aligners into components favorable to the desired movement. The decomposition of a force applied to an inclined plane results in a normal force component, which enables the production of movements in directions other than the buccolingual axis. 

A precise understanding of attachments can prevent their unnecessary use in cases in which benefits do not justify the risks, as their bonding requires acid etching, which alters the structure of healthy enamel and may increase the likelihood of plaque retention, formation of white spot lesions^29^, cavities, and introduces risks during their removal^30^. Therefore, attachments should only be added if they have a clear and necessary function. 

The primary function of attachments is to create a surface with an angle that allows modification of the normal force direction according to the desired tooth movement, also altering its magnitude. A secondary function that may be attributed to attachments is increasing aligner retention, when needed. 

There is a wide variety of attachment designs, offering many options for each clinical condition. The most common shapes are single or double ellipsoids and rectangles, sometimes featuring a beveled surface-that is, an inclined plane designed to favor the direction and intensity of the force. These catalogs may be considered irrelevant if one understands that regardless of their shape, to assist in movement, there will always be a contact surface. Broadly speaking, attachments can be classified into just two types: movement attachments and anchorage attachments. 

Movement attachments have the clear function of redirecting the force exerted by the aligner. As previously discussed, aligners are activated when they open their internal gap upon being fitted into the mouth and deactivate when they return to their original shape. At that point, only forces in the buccolingual direction are generated. Therefore, if it is necessary to apply forces in other directions-for example, vertically relative to the occlusal plane-, and those forces must be redirected toward the desired movement. 

As previously discussed, the stiffness of the aligner material under tensile forces results in force levels too high for orthodontic applications, with minimal deformation. This characteristic explains the greater difficulty in performing vertical movements with this type of appliance^31^. Therefore, to explain the action of aligners with attachments, we use extrusion movement as an example, which later simplifies the understanding of other movements that can be achieved simply by redirecting the contact surface of the attachment. 

Adding attachments to the extrusion plan of teeth will interfere with the aligner’s closure during seating, preventing complete adaptation and reducing the contact area between the aligner and teeth-contrary to common belief (Fig. 17). 

**Figure 16:**
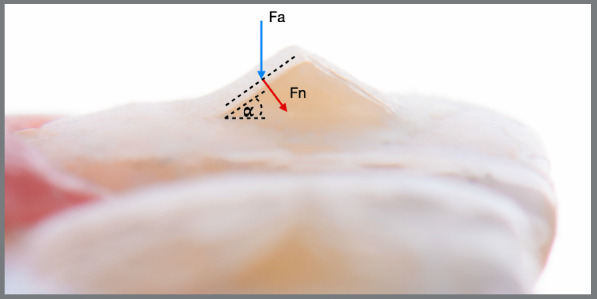
Force Fa applied by the aligner is intercepted by the inclined plane. The normal force Fn, always perpendicular to the contact surface, has its new direction determined by the plane’s angle (α).

**Figure 17:**
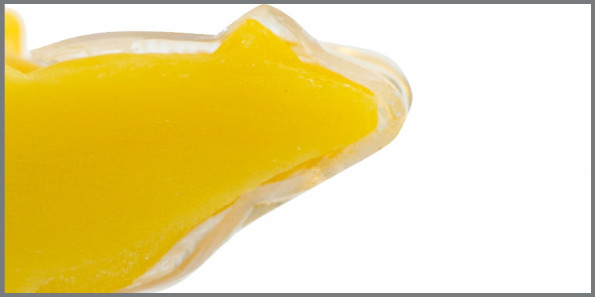
Aligner with programmed extrusion movement contacts only the attachment and loses contact with the entire tooth surface until movement occurs.

When the goal is to extrude a tooth, the inclined plane should have its active surface facing cervically, higher toward the incisal/occlusal side of the tooth (Fig. 16). The lingual force F generated by the closure of the aligner’s buccal surface is decomposed on the inclined plane, and the useful force for extrusion increases from zero to a fraction of the initial force, depending on the angle of the plane. 

The attachment surface and the internal surface of the aligner must be as smooth as possible, to prevent excessive friction, which could impair tooth movement. In aligners, especially when associated with beveled attachments, displacement occurs by sliding. Thus, the normal force responsible for the desired movement also contributes to friction. 

Salivary fluid helps reduce friction by acting as a lubricant, as does the proper polishing of the attachment surface. This polishing depends on the precision of the bonding guide, the quality of the resin used, and continuous monitoring of its integrity at each appointment. The smoothness of the aligner’s inner surface is directly related to the precision of printed models, the quality of the thermoforming process, and the tooth crown anatomy. 

**Figure 18:**
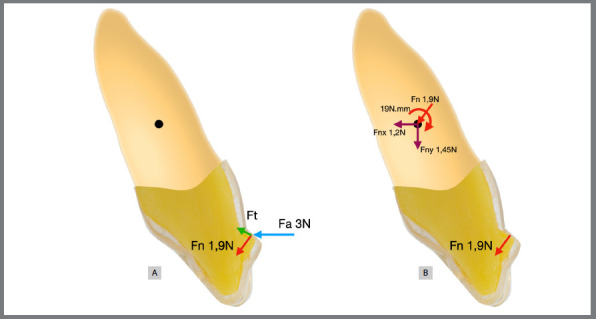
A) A 3N force applied to an attachment with 50° inclination results in a 1.9N normal force and tangential component Ft. B) Normal force transferred to the center of resistance, with a 19 N·mm moment, decomposing Fn into Fnx (1.2N) and Fny (1.45N).

If the aligner applies a force of 3N to an attachment oriented for extrusion movement (Fig. 18), with an active surface inclined at approximately 50° relative to the occlusal plane, the resulting normal force will be 1.9N (Fn = Fa × cos θ). 

By transferring the force system applied on the attachment to the tooth’s center of resistance, we can better understand the type of movement expected from the forces applied by orthodontic devices. In Figure 18, the extrusive force component (Fny) represented at the center of resistance would be approximately 1.45N for a 3N force applied to the extrusion attachment. However, the effective extrusive force could be even lower, as we must subtract the likely frictional force generated by contact between the tooth’s lingual surface and the aligner. Besides the extrusive force derived from the inclined plane, the normal force remains directed lingually, and both contribute to lingual tipping of the tooth. From this analysis, it becomes clear why extrusion plans with lingual inclination of the teeth are more successful than those aiming for extrusion along the long axis, where tipping is blocked. All quantitative measurements are instantaneous, as these forces are known to degrade by about 60% within the first few hours of aligner use^23^. 

Therefore, in any situation where there is a need to redirect the force generated by the aligner, movement attachments (inclined plane) can be used. 

The contact points between the aligner and the tooth to be moved will determine the force’s line of action, which will tend to cause a displacement associated with tipping. 

When using extrusion attachments on the buccal surface of teeth, it is important to remember that their effectiveness is related to the possibility of displacement in the direction of the force and palatal tipping. If the tooth is not meant to tip and this movement is not included in the setup, collision with the aligner on the palatal side will block the tipping effect and hinder the desired extrusion movement. 

The attachment’s name or model is irrelevant to its clinical function. What truly matters is its placement and the orientation of the inclined plane. Therefore, attachments intended for tooth movement could maintain a standardized shape, provided they are adapted to the tooth size and the direction of the force required for the desired movement. 

## CONCLUSIONS

Understanding how aligners are activated, the importance of the incisal edge in the initial contact, the deactivation of the buccal surface on the teeth, and how to intercept the deactivation force through the use of attachments can facilitate the clinical use of aligners and aid in the development of research that more closely reflects actual tooth movements. In their current configuration, aligners are not suitable for generating orthodontic forces involving other types of loading, such as traction, torsion, and shear in the buccal and lingual planes, making them inadequate for movements such as traction, mesiodistal angulations, or vertical deformations. However, the outcome of orthodontic movement involves not only the physical and mechanical aspects of orthodontic appliances but also biological factors such as bone structure and density, the anisotropy of the periodontal ligament, and systemic factors, which are beyond the scope of this analysis. Other movements, such as buccal tipping or proximal shifts of anterior teeth, can be understood in the same manner described in this article, but torque, rotations, and proximal movements in posterior teeth are achieved by different mechanisms and were not addressed in this study. 

## Data Availability

All data generated or analyzed during this study are included in this published article
